# Patient-specific core decompression surgery for early-stage ischemic necrosis of the femoral head

**DOI:** 10.1371/journal.pone.0175366

**Published:** 2017-05-02

**Authors:** Wei Wang, Wei Hu, Pei Yang, Xiao Qian Dang, Xiao Hui Li, Kun Zheng Wang

**Affiliations:** 1 The First Department of Orthopaedics, The Second Affiliated Hospital of Xi'an Jiaotong University, Xi'an, Shaanxi Province, P. R. China; 2 The Department of Orthopaedics, Xian Yang Central Hospital, Xian Yang, Shaanxi Province, P. R. China; 3 Radiology Department, The Second Affiliated Hospital of Xi'an Jiaotong University, Xi'an, Shaanxi Province, P. R. China; University of Umeå, SWEDEN

## Abstract

**Introduction:**

Core decompression is an efficient treatment for early stage ischemic necrosis of the femoral head. In conventional procedures, the pre-operative X-ray only shows one plane of the ischemic area, which often results in inaccurate drilling. This paper introduces a new method that uses computer-assisted technology and rapid prototyping to enhance drilling accuracy during core decompression surgeries and presents a validation study of cadaveric tests.

**Methods:**

Twelve cadaveric human femurs were used to simulate early-stage ischemic necrosis. The core decompression target at the anterolateral femoral head was simulated using an embedded glass ball (target). Three positioning Kirschner wires were drilled into the top and bottom of the large rotor. The specimen was then subjected to computed tomography (CT). A CT image of the specimen was imported into the Mimics software to construct a three-dimensional model including the target. The best core decompression channel was then designed using the 3D model. A navigational template for the specimen was designed using the Pro/E software and manufactured by rapid prototyping technology to guide the drilling channel. The specimen-specific navigation template was installed on the specimen using positioning Kirschner wires. Drilling was performed using a guide needle through the guiding hole on the templates. The distance between the end point of the guide needle and the target was measured to validate the patient-specific surgical accuracy.

**Results:**

The average distance between the tip of the guide needle drilled through the guiding template and the target was 1.92±0.071 mm.

**Conclusions:**

Core decompression using a computer-rapid prototyping template is a reliable and accurate technique that could provide a new method of precision decompression for early-stage ischemic necrosis.

## Introduction

Core decompression surgery, which was first proposed by Ficat and Arlet in 1964, is used to treat early-stage ischemic necrosis of the femoral head to alleviate pain, to reduce the pressure in the internal medullary cavity of the femoral head, to improve local blood circulation, and to delay femoral head replacement due to femoral head necrosis collapse and loss of hip joint function [1–3]. Since its implementation, core decompression surgery has evolved from simple core decompression to multiple core decompression [4]. Nevertheless, despite its widespread use, there are no studies on the precise location of the ischemic areas before and during surgery or on acquiring three-dimensional (3D) information of the ischemic areas. Information on the ischemic areas are still obtained using X-ray, computed tomography (CT), and magnetic resonance imaging (MRI), all of which are two-dimensional (2D) techniques. Surgery is still guided by C-arm X-ray using 2D data to locate the puncture position.

Iatrogenic injuries are more likely to occur after multiple drillings, for which more important X-ray exposure is required. Such damage and inaccurate drilling may result in variable success rates and some studies even considered that core compression was useless for osteonecrosis of the femoral head [5–9]. Finding a way to improve the precision of necrotic area locations should reduce the damage caused by multiple punctures and X-ray exposure and the success rate should increase. With the development of computer-assisted orthopedic surgery, computer-assisted techniques are more and more widely applied in high-precision orthopedic surgeries [10–12].

This study, which is based on computer-assisted rapid prototyping, was designed to simulate core decompression of a femur containing locating wires and to use a rapid prototyping technology to manufacture an individual navigation template adapted to the locating wires for core decompression surgery. Here we describe a precise core decompression procedure for treating early-stage osteonecrosis of the femoral head that not only determine the accurate location of the ischemic areas, but also reduces the damage caused by traditional core decompression and slow down the progression of femoral head necrosis.

## Materials and methods

### Specimen preparation

Twelve adult fresh femurs (Department of Anatomy, Xi’an Jiaotong University) were used in this study. These femurs were teaching bones used at the Department of Anatomy of Xi’an Jiaotong University (see S1 File). All body parts were anonymized upon harvesting. This study was approved by the ethical committee of the Second Hospital affiliated to Xi’an Jiaotong University (see S2 File). Causes of death were various and were not associated with the body parts. Only intact femurs were used. None of the tissue donors were from a vulnerable population and all donors or next of kin provided written informed consent for the use of the cadavers for teaching and research purposes.

**Fig 1:**
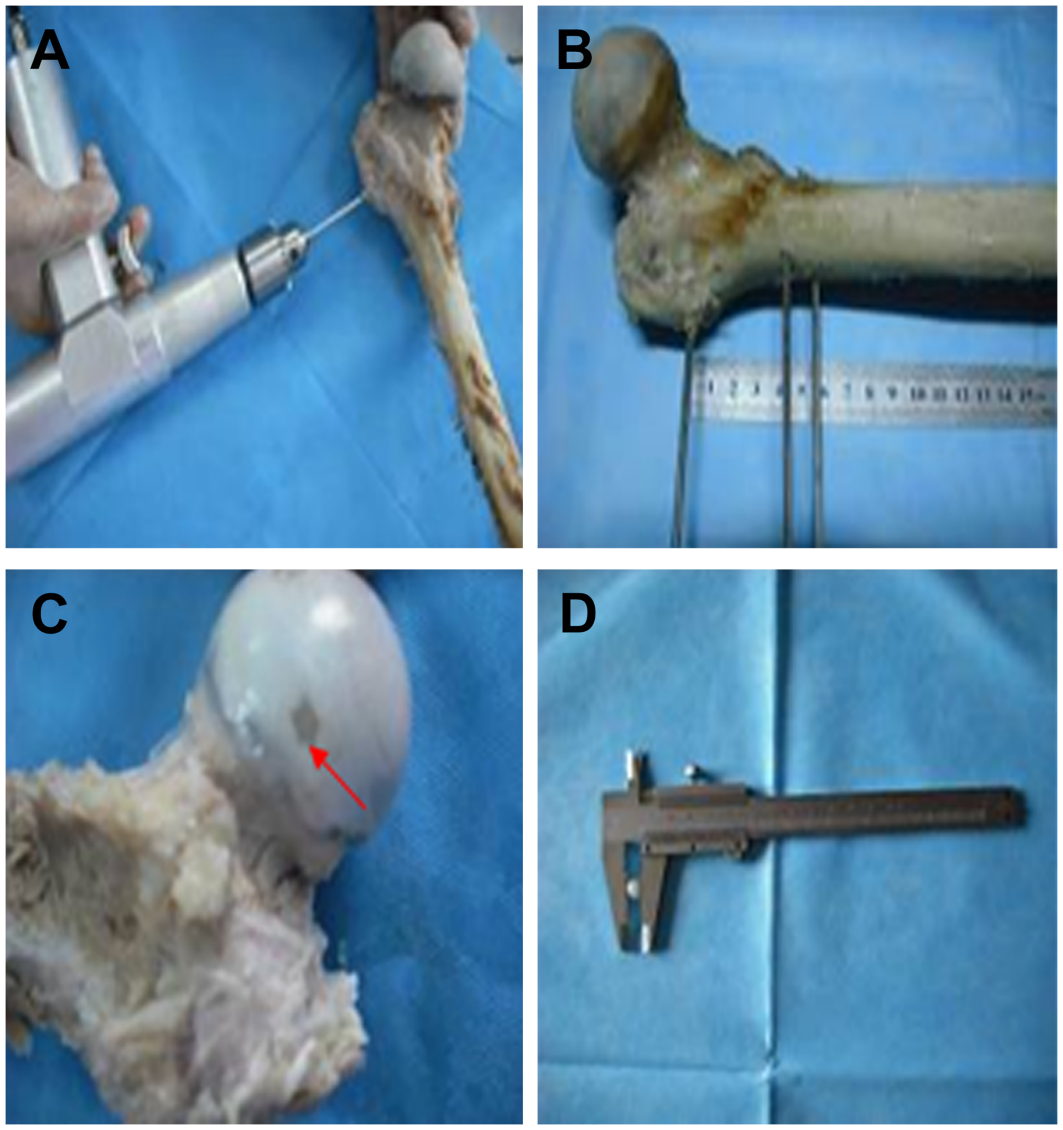
Specimen preparation. Three positioning Kirschner wires (3 mm in diameter, 100 mm long) were fixed into the top greater trochanter of the femur, as well as 40- and 60-mm below it. A 5-mm glass ball was placed in the femoral head as the target spot for decompression. A. Fixation of the Kirschner wires into the femur. B. The femoral specimen with the three Kirschner wires. C. The glass ball into the right upper quadrant of the femoral head and sealed with bone wax. D. The 5-mm glass ball.

Three positioning Kirschner wires (3 mm in diameter, 100 mm long) were fixed into the top greater trochanter of the femur, as well as 40- and 60-mm below it. A 5-mm glass ball was placed in the femoral head as the target spot for decompression (Fig 1A–1D).

**Fig 2:**
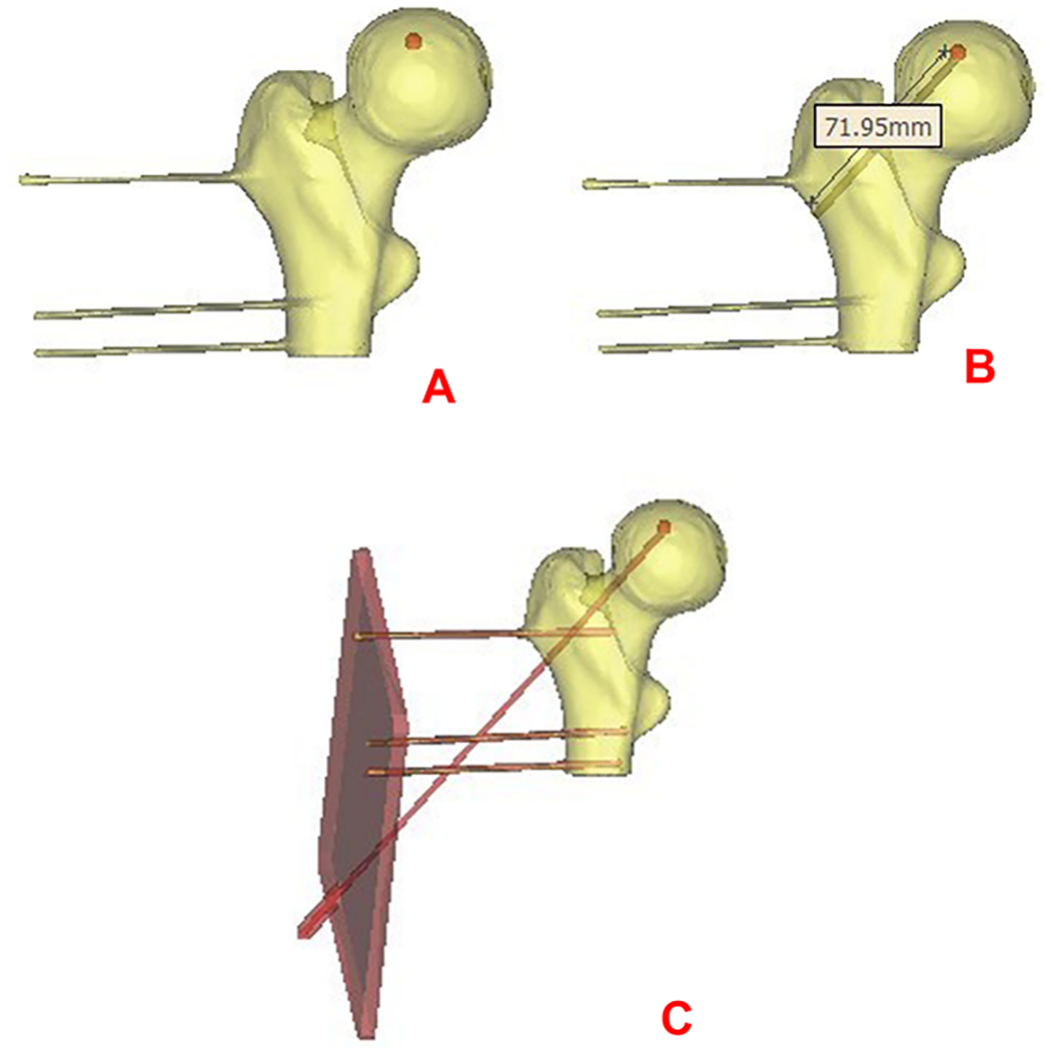
The reconstructed specimen model was smoothed, core decompression was digitally simulated, and navigation templates were designed. A. The reconstructed specimen model was smoothed and the glass ball was marked in red in the picture. B. The depth of the osseous part of the core decompression channel was measured, and all data were saved in STL format. C. A template with four holes (3 mm in diameter) was created. Three holes were adapted to the positioning wires on the specimen, and the fourth hole was adapted to the drilling direction of the guide needle for the core decompression.

The images were collected and the 3-D models were reconstructed. A spiral 64-slice CT (Lightspeed vct 64 CT; GE Healthcare, Waukesha, WI, USA) was used to scan the prepared specimen (0.625-mm slice thickness, 120 kV, 240 mA). The data were saved in DICOM format and imported into the Mimics 10.01 software (Materialise, Leuven, Belgium). In Mimics, there were 119 layers in the windows of the coronal section and 89 layers in the windows of the transverse section. Each layer mask was compiled to fill in the blanks and high-quality reconstruction parameters were set to calculate the 3D model of the femurs. The reconstructed specimen model was smoothed thereafter. The masks of the layers of the target spot, which included 10 layers on the coronal section and 6 layers on the transverse section, were compiled to reconstruct the 3D model of the glass ball, and then the reconstructed specimen model was smoothed(Fig 2A).

### Digital simulation of core decompression

The segmentation module in Mimics was used to calculate the profile curve of the femoral head, and a sphere was then designed according to the profile curve. The center coordinates of this sphere were considered as the center coordinate of the femoral head. The first layer of the glass ball on the coronal section was considered as the target for core decompression, and its coordinates were calculated. The center coordinates of the femoral head were considered as the origin to calculate the 3D coordinates of the target spot in the core decompression. Under semitransparent visualization of the Simulation module of Mimics, the spot that was 20 mm below the top of the greater trochanter of the femur was considered as the starting point of the core decompression, while the first layer of the glass ball on the coronal section was considered as the finishing point of the core decompression channel. A core decompression channel of 3 mm in diameter was designed on the basis of these two points. The depth of the osseous portion of the core decompression channel was measured and all of the data above were saved in STL format (Fig 2B).

The data of the femoral specimens with the designed core decompression channel were imported into the Pro/E software (PTC, Needham, MA, USA) in STL format. A navigation template was designed to adapt the positioning wires on the specimen. It was also adapted to a guide rod that fit the core decompression channel. A four-hole template (3 mm in diameter) was created. Three of these holes were adapted to the positioning wires on the specimen, while the fourth hole was adapted to the drilling direction of the guide needle for core decompression (Fig 2C). The dimensions of the navigation template were 120-mm long, 80-mm wide, and 5-mm thick, and the distance between the guide plate and the bonce was 50 mm. All data were imported into a rapid prototyping machine, and the laser sintering method was chosen to mold the light-sensitive resin, which was then solidified and added to the navigation template.

**Fig 3:**
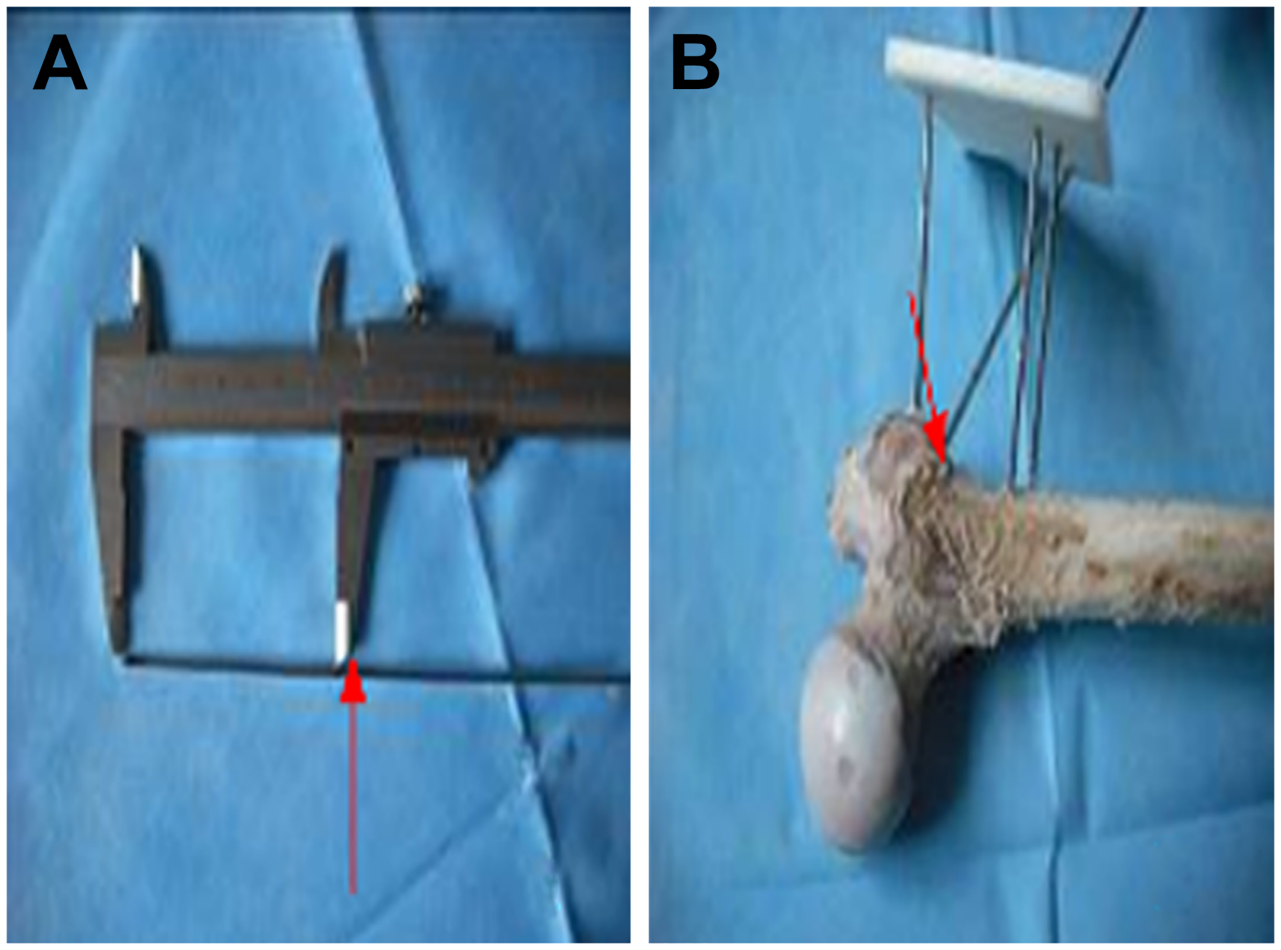
Use of the navigation template for core decompression. A. The drilling depth was based on the measurement of the core decompression’s osseous channel. B. The navigation template was placed on the three positioning Kirschner wires at a distance of 50 mm from the bone, and another Kirschner wire (3 mm in diameter) marked with decompression depth was drilled into the femoral head through the guide rod direction.

The navigation template was placed stably on the three positioning Kirschner wires and another Kirschner wire (3 mm in diameter) marked with decompression depth was drilled into the femoral head through the guide rod direction. The drilling depth was based on the measurement of the core decompression’s osseous channel (Fig 3A and 3B).

After drilling, the femoral head specimen with the core decompression guide needle was scanned with CT. The CT data were saved in DICOM format and imported into Mimics. The 3D model of the femoral specimen with the decompression wire was established after layer mask editing, cavity filling, and smoothing. The coordinates of the decompression needle end were calculated, and the range error between the needle end and the target spot was measured according to this coordinate and that of the first layer of the glass ball on the coronal section.

## Results

### Reconstruction

Using the CT data, 3D reconstruction required approximately 30 min. The computer simulated surgery required about 90 min. The printing of the navigation template required about 180 min. Therefore, the whole process took about 5 h. The price of each navigation template was about 100 yuan (about 17 USD).

### Center coordinates

After the reconstruction by computer, the glass ball was considered as a simulated necrotic lesion center. Table 1 shows the exact location in the femoral head in the simulated necrotic lesion center of each specimen.

**Table 1:** The center coordinates of the femoral head as the reference; the glass ball was located in 3D.

No.	Center coordinates of the femoral head (x, y, z)	Center coordinates of the glass ball (x, y, z)	Center coordinates of the glass ball when the center of the femoral head is taken as the origin (x, y, z)
1	(122.54,140.43,142.76)	(102.35,131.50,155.49)	(-20.19,-8.93,12.37)
2	(174.63,152.64,148.16)	(175.75,144.43,158.24)	(1.12, -8.12, 10.08)
3	(142.10,182.70,234.11)	(148.65,175.84,246.55)	(6.55, -6.86, 12.44)
4	(200.38,183.38,237.39)	(197.11,174.47,247.86)	(-3.27, -8.91,10.47)
5	(139.32,171.27,203.28)	(121.21,165.24,217.45)	(-18.1, -6.03,14.26)
6	(162.54,170.41,210.14)	(159.31,158.42,216.69)	(-3.23, -11.99,6.55)
7	(129.84,191.61,149.32)	(117.12,188.50,157.43)	(-12.72, -3.01,8.11)
8	(183.71,201.37,199.94)	(172.38,198.23,217.54)	(-11.33, -3.14,17.6)
9	(157.24,183.64,183.74)	(141.93,178.46,196.54)	(-15.31, -5.18,12.8)
10	(175.35,193.74,200.18)	(168.91,179.31,211.65)	(-6.44,-14.43,11.47)
11	(146.82,172.63,189.07)	(137.51,169.87,201.61)	(-9.31, -2.76,12.54)
12	(184.11,198.42,217.13)	(169.61,189.03,226.41)	(-14.50, -9.39,9.28)

### Drilling accuracy

**Fig 4:**
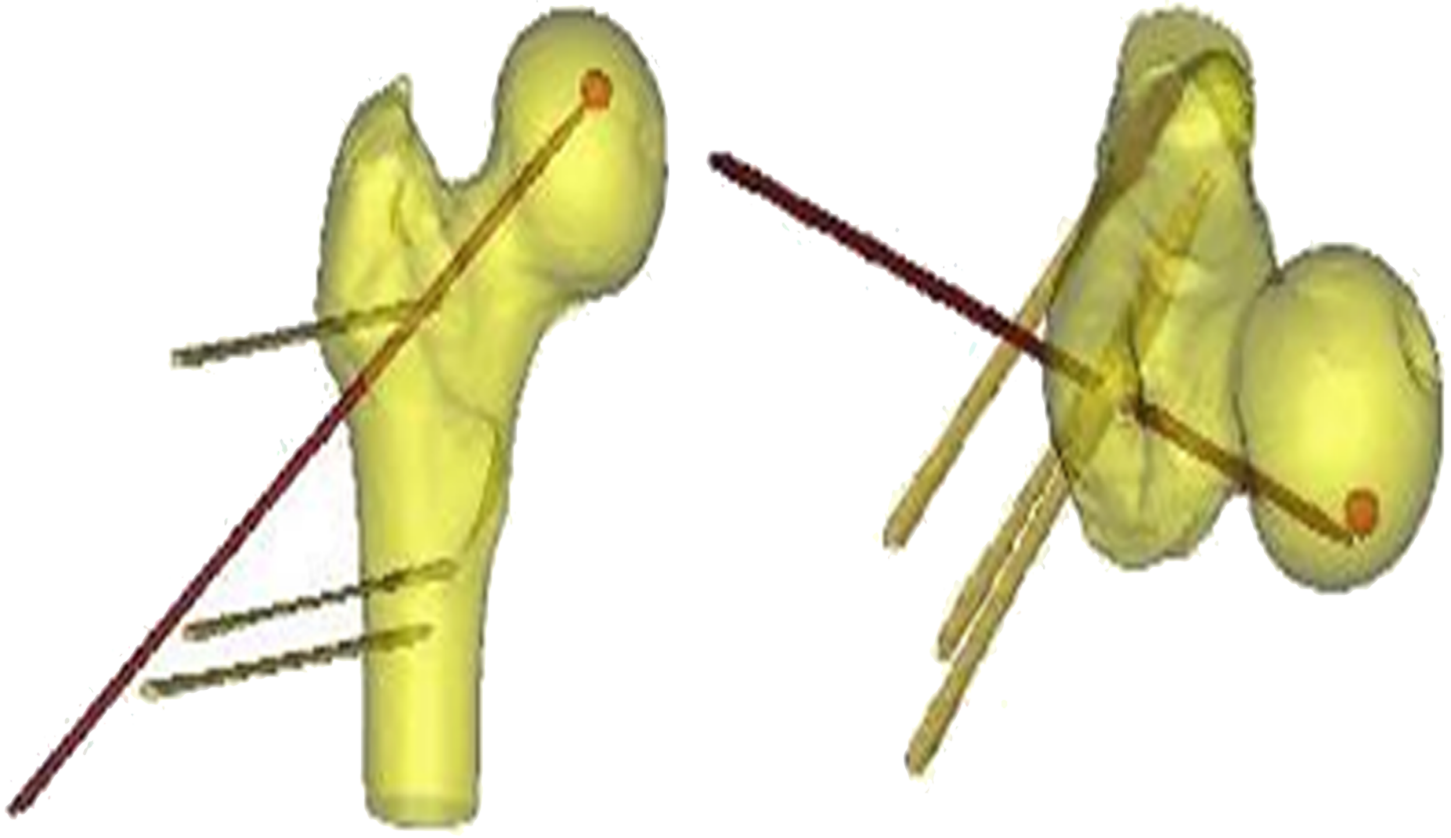
The reconstructed model of femoral specimen and the guide needle (anterior aspect and superior aspect).

After drilling, the CT scan data were imported into Mimics, and a model was built (Fig 4A and 4B). The distance between the target spot and the destination was measured of the coronal section in Mimics, and the range error between them was calculated. The calculated average range error was 1.92±0.071 mm (Table 2).

**Table 2:** Range error (mm) between the target and the needle’s end.

No.	ΔX	ΔY	ΔZ	ΔL
1	1.58	1.07	- 0.32	1.94
2	- 1.47	1.11	0.27	1.86
3	1.02	0.98	1.13	1.81
4	1.63	- 1.12	0.19	1.99
5	0.57	1.26	- 1.49	2.03
6	1.37	1.18	0.21	1.82
7	1.06	- 1.51	0.39	1.89
8	- 1.25	0.42	1.46	1.97
9	1.18	- 0.53	1.38	1.89
10	- 1.48	1.17	0.43	1.93
11	0.51	- 1.29	1.46	2.01
12	1.47	1.15	0.47	1.92

## Discussion

Early diagnosis and treatment is crucial for improving long-term prognosis of ischemic necrosis of the femoral head [13,14]. Much research has shown that precise core decompression effectively treats ischemic necrosis in Ficat I and Ficat II [13–15]. Accuracy is essential during core decompression [16], but conventional techniques use preoperative 2D data such as X-ray, CT, and MRI, which makes it difficult to precisely locate the irregular ischemic areas. As a result, multiple drillings and depths often have to be used, increasing radiation exposure for both the patient and surgical team as well as increasing the risk of iatrogenic cartilage and bone fracture [17]. Multiple drilling also means a longer surgery and greater risks of infection and surgical complications.

Computer-assisted imaging techniques are currently used in an effort to increase surgical accuracy. These new techniques enable computer-assisted orthopedic surgery (CAOS), in which CT and MRI scan data are transferred into 3D representations that guide surgeons during the operation. Because CAOS provides much higher precision and control than conventional techniques, it has been widely used in various kinds of orthopedic surgeries [11,18,19]. Some types of CAOS provide a navigation system that shows simultaneous coronal and sagittal plane views on the system-associated screen during surgery [16,20–23]. Drilling is then performed by the real-time visualization of targeting the ischemic areas on the screen. These navigational systems promise a high accuracy and reduction of radiation exposure time, but it is still limited by the lengthy registration process. Moreover, the inevitable steep learning curve and device expenses hamper the implementation of these navigational systems in less developed areas [24].

Surgical improvements are provided by the 3D digital reconstruction and reverse engineering techniques. A promising prospect has been shown in clinical application [9,10]. The error range can be controlled to 0.1 mm [25]. This study evaluated the feasibility of the use of rapid prototyping for femoral head core decompression. Rapid prototyping can provide a clearer and 3D view of the skeletal anatomy to the orthopedist [26,27]. Our approach is that after K-wire fixation and CT scan at the primary hospital, the relevant imaging data can be sent to a more important hospital or 3D printing center, where the navigation template can be accurately manufactured and mailed to the primary hospital. The surgery can then be performed at the primary hospitals according to the navigation template. Therefore, compared with the navigation systems, it has the advantages of low cost, no need for intraoperative registration, decreased learning curve, and shortened surgery time. In addition, surgery can be preoperatively previewed to ensure the accurate location. The operation is performed without the need to estimate the target area or use fluoroscopy and C-arm X-ray, which reduces the surgical complexity and the exposure to radiations. Most importantly, a very short learning curve is required for this technique since it can easily be mastered by any surgeon who is familiar with the core decompression procedure.

In this study, the distance between the navigation template and the proximal femur was fixed at 50 mm, but considering the eventual clinical applications in the future, the distance between the navigation template and the proximal femur will be individually designed according to the degree of obesity and local thickness of soft tissue among different patients. These parameters will be taken into account using the CT images in the Mimics software. This distance would not affect the accuracy of the navigation since they will have been taken into account when designing the plate.

Nevertheless, this novel approach may have some disadvantages and the following errors may occur: manually slicing the graph and reconstructing the model, mismatch between the rapid prototype model and the 3D model in the computer, navigational template location, and slight movement of the template during the procedure. Despite these errors, there was a 1.92-mm range error between the needle’s end and the target’s center coordinates since the necrotic areas can be large, which is considered to match the clinical requirement of core decompression. In addition, the use of three K-wires for template alignment near the core decompression site could the fracture risk. Since the current experiment presents only ex vivo conditions, its data and results cannot be directly extrapolated to in vivo circumstances. In addition, including a control group should be ideal. However, the present study was only a pilot proof-of-concept study that allowed showing the possibility of using the Mimics software and rapid prototyping to achieve high drilling accuracy. Additional studies are necessary before implementation in the clinical setting and a control group will then be included. In addition, a reproducibility study of the influence of the horizontality of K-wire drilling is necessary.

In conclusion, core decompression using a computer-rapid prototyping template is a reliable and accurate technique that could provide a new method of precision decompression for early-stage ischemic necrosis.

## Supporting information

S1 File Supplement document. (PDF)

S2 File English ethnical document. (JPG)

## References

[1] Lieberman JR. Core decompression for osteonecrosis of the hip. Clin Orthop Relat Res. 2004: 29–33. 10.1097/00003086-200401000-00006 15043089

[2] Mont MA, Jones LC, Hungerford DS. Nontraumatic osteonecrosis of the femoral head: ten years later. J Bone Joint Surg Am. 2006;88: 1117–1132. 10.2106/JBJS.E.01041 16651589

[3] van der Jagt D, Mokete L, Pietrzak J, Zalavras CG, Lieberman JR. Osteonecrosis of the femoral head: evaluation and treatment. J Am Acad Orthop Surg. 2015;23: 69–70. 10.5435/JAAOS-D-14-00431 25624358

[4] Song WS, Yoo JJ, Kim YM, Kim HJ. Results of multiple drilling compared with those of conventional methods of core decompression. Clin Orthop Relat Res. 2007;454: 139–146. 10.1097/01.blo.0000229342.96103.73 16906081

[5] Castro FP Jr., Barrack RL. Core decompression and conservative treatment for avascular necrosis of the femoral head: a meta-analysis. Am J Orthop (Belle Mead NJ). 2000;29: 187–194. 10746469

[6] Fairbank AC, Bhatia D, Jinnah RH, Hungerford DS. Long-term results of core decompression for ischaemic necrosis of the femoral head. J Bone Joint Surg Br. 1995;77: 42–49. 7822394

[7] Yu PA, Peng KT, Huang TW, Hsu RW, Hsu WH, Lee MS. Injectable synthetic bone graft substitute combined with core decompression in the treatment of advanced osteonecrosis of the femoral head: A 5-year follow-up. Biomed J. 2015;38: 257–261. 10.4103/2319-4170.138307 25179724

[8] Lieberman JR, Engstrom SM, Meneghini RM, SooHoo NF. Which factors influence preservation of the osteonecrotic femoral head? Clin Orthop Relat Res. 2012;470: 525–534. 10.1007/s11999-011-2050-4 21879405 PMC3254748

[9] Lu S, Xu YQ, Zhang YZ, Li YB, Xie L, Shi JH, et al A novel computer-assisted drill guide template for lumbar pedicle screw placement: a cadaveric and clinical study. Int J Med Robot. 2009;5: 184–191. 10.1002/rcs.249 19280584

[10] Zhang YZ, Chen B, Lu S, Yang Y, Zhao JM, Liu R, et al Preliminary application of computer-assisted patient-specific acetabular navigational template for total hip arthroplasty in adult single development dysplasia of the hip. Int J Med Robot. 2011;7: 469–474. 10.1002/rcs.423 22113980

[11] Sidon E, Steinberg EL. Accuracy study of new computer-assisted orthopedic surgery software. Eur J Radiol. 2012;81: 4029–4034. 10.1016/j.ejrad.2012.07.016 22883531

[12] Vlamis J, Karampinas P, Kavroudakis E, Pneumaticos S. The use of core track endoscopy to document accurate decompression of the femoral head. Hip Int. 2014;24: 284–289. 24500830 10.5301/hipint.5000118

[13] Soucacos PN, Beris AE, Malizos K, Koropilias A, Zalavras H, Dailiana Z. Treatment of avascular necrosis of the femoral head with vascularized fibular transplant. Clin Orthop Relat Res. 2001: 120–130. 11347825 10.1097/00003086-200105000-00016

[14] Steinberg ME, Larcom PG, Strafford B, Hosick WB, Corces A, Bands RE, et al Core decompression with bone grafting for osteonecrosis of the femoral head. Clin Orthop Relat Res. 2001: 71–78. 10.1097/00003086-200105000-00009 11347851

[15] Aigner N, Schneider W, Eberl V, Knahr K. Core decompression in early stages of femoral head osteonecrosis—an MRI-controlled study. Int Orthop. 2002;26: 31–35. 10.1007/s00264-001-0311-7 11954846 PMC3620848

[16] Beckmann J, Goetz J, Baethis H, Kalteis T, Grifka J, Perlick L. Precision of computer-assisted core decompression drilling of the femoral head. Arch Orthop Trauma Surg. 2006;126: 374–379. 10.1007/s00402-006-0155-0 16738924

[17] Pierannunzii L. Endoscopic and arthroscopic assistance in femoral head core decompression. Arthrosc Tech. 2012;1: e225–230. 10.1016/j.eats.2012.08.004 23767000 PMC3678640

[18] Schep NW, Broeders IA, van der Werken C. Computer assisted orthopaedic and trauma surgery. State of the art and future perspectives. Injury. 2003;34: 299–306. 12667784 10.1016/s0020-1383(01)00208-x

[19] Kahler DM. Image guidance: fluoroscopic navigation. Clin Orthop Relat Res. 2004: 70–76. 15123929

[20] Mayman D, Vasarhelyi EM, Long W, Ellis RE, Rudan J, Pichora DR. Computer-assisted guidewire insertion for hip fracture fixation. J Orthop Trauma. 2005;19: 610–615. 16247305 10.1097/01.bot.0000177106.30837.28

[21] Khoury A, Liebergall M, Weil Y, Mosheiff R. Computerized fluoroscopic-based navigation-assisted intramedullary nailing. Am J Orthop (Belle Mead NJ). 2007;36: 582–585. 18075604

[22] Mosheiff R, Weil Y, Peleg E, Liebergall M. Computerised navigation for closed reduction during femoral intramedullary nailing. Injury. 2005;36: 866–870. 10.1016/j.injury.2004.12.036 15949490

[23] Weil YA, Liebergall M, Mosheiff R, Helfet DL, Pearle AD. Long bone fracture reduction using a fluoroscopy-based navigation system: a feasibility and accuracy study. Comput Aided Surg. 2007;12: 295–302. 10.3109/10929080701657974 17957537

[24] Sikorski JM, Chauhan S. Computer-assisted orthopaedic surgery: do we need CAOS? J Bone Joint Surg Br. 2003;85: 319–323. 12729101 10.1302/0301-620x.85b3.14212

[25] Zhang YZ, Lu S, Yang Y, Xu YQ, Li YB, Pei GX. Design and primary application of computer-assisted, patient-specific navigational templates in metal-on-metal hip resurfacing arthroplasty. J Arthroplasty. 2011;26: 1083–1087. 10.1016/j.arth.2010.08.004 20932709

[26] Brown GA, Firoozbakhsh K, DeCoster TA, Reyna JR Jr., Moneim M. Rapid prototyping: the future of trauma surgery? J Bone Joint Surg Am. 2003;85-A Suppl 4: 49–55. 14652393

[27] Pham AM, Rafii AA, Metzger MC, Jamali A, Strong EB. Computer modeling and intraoperative navigation in maxillofacial surgery. Otolaryngol Head Neck Surg. 2007;137: 624–631. 10.1016/j.otohns.2007.06.719 17903581

