# Supplementary figures and images for: Patient-specific core decompression surgery for early-stage ischemic necrosis of the femoral head
Source: PLoS One. 2017 May 2;12(5):e0175366. doi: 10.1371/journal.pone.0175366 (PMC5413021; doi:10.1371/journal.pone.0175366)

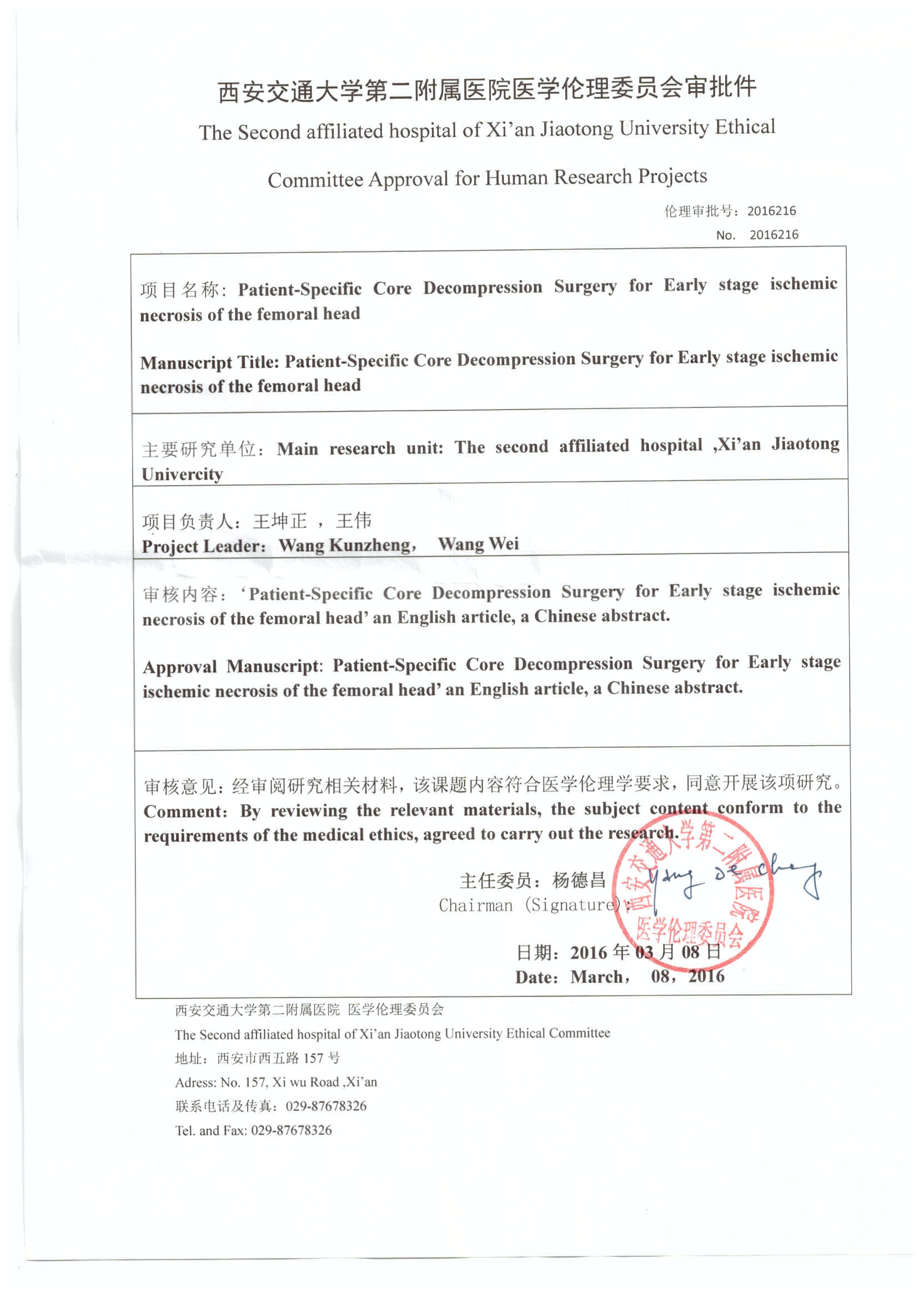

Supplement: S2 File — (JPG) [file pone.0175366.s002.jpg]
