# Supplementary material for: Patient-specific core decompression surgery for early-stage ischemic necrosis of the femoral head
Source: PLoS One. 2017 May 2;12(5):e0175366. doi: 10.1371/journal.pone.0175366 (PMC5413021; doi:10.1371/journal.pone.0175366)
Supplement: S1 File — (PDF) [file pone.0175366.s001.pdf]

Dear editor of PLOS One:

We declared that the donated tissue of the department of anatomy in xi’an Jiaotong university were not from a vulnerable population and all donors or next of kin provided written informed consent that was freely given to teaching or scientific research. However, we can't provide informed consent due to the privacy of donors. In addition, the cause of death for bone donor was not linked to the osteopathy.

Materials of femurs in manuscript (Patient-Specific Core Decompression Surgery for Early stage ischemic necrosis of the femoral head (No:PONE-D-16-23609) were from above tissue donors. We agree with professor Wang used these femurs in scientific research.

Best wishes!

西安交通大学医学中心 遗体捐献工作服务办公室

The body donation service office of Xi 'an jiaotong university medical center
